# Impact of donor organ quality on recipient outcomes in lung transplantation: 14-Year single-center experience using the Eurotransplant lung donor score

**DOI:** 10.1016/j.jhlto.2024.100166

**Published:** 2024-10-11

**Authors:** Katharina Flöthmann, Nunzio Davide de Manna, Khalil Aburahma, Sophie Kruszona, Philipp Wand, Dmitry Bobylev, Carsten Müller, Julia Carlens, Nicolaus Schwerk, Murat Avsar, Arjang Ruhparwar, Christian Kühn, Mark Greer, Jawad Salman, Fabio Ius

**Affiliations:** a Department of Cardiothoracic, Transplant and Vascular Surgery, Hannover Medical School, Hannover, Germany; b Department of Pediatric Pneumology, Allergology and Neonatology, Hannover Medical School, Hannover, Germany; c Department of Respiratory Medicine and Infectious Diseases, Hannover Medical School, Hannover, Germany; d German Center for Lung Research (DZL/BREATH), Hannover, Germany

**Keywords:** lung transplantation, donor quality, Eurotransplant score, extended-criteria lung donor

## Abstract

**Background:**

The use of extended-criteria donor (ECD) organs has increased in lung transplantation, but their impact on long-term outcomes remains unclear. This retrospective single-center study evaluates the impact of donor quality, as defined by the Eurotransplant (ET) lung donor score, on long-term graft function and survival.

**Methods:**

Records of recipients transplanted between January 2010 and May 2023 were reviewed. Eurotransplant lung donor scores (ET scores) were retrospectively calculated from the corresponding donor reports. Outcomes were compared between recipients of donor lungs with an ET score of 6 (group 1), 7 and 8 (group 2), and 9 to 13 (group 3, ECD lungs). Median follow-up was 64 (30-104) months.

**Results:**

In total, 280 (19%) patients were transplanted with ET score 6 lungs, 717 (48%) patients with ET scores 7 and 8 lungs, and 506 (34%) patients with ET scores 9 to 13 (ECD) lungs. The occurrence of primary graft dysfunction grade 3 at 72 hours (*p* = 0.672), duration of mechanical ventilation (*p* = 0.062), and in-hospital mortality (*p* = 0.713) did not differ between groups. Long-term graft survival (%) was lower in group 2 and 3 vs group 1 recipients (at 10 years: 51 and 48 vs 56, *p* = 0.052, respectively). Similarly, freedom from chronic lung allograft dysfunction (CLAD, %) was lower in group 2 and 3 vs group 1 recipients (at 10 years: 57 and 55 vs 63, *p* = 0.033, respectively). Donor smoking history was associated with worse CLAD-free survival (hazard ratio = 1.466, 95% confidence interval = 1.215-1.769, *p* < 0.001).

**Conclusions:**

ECD lungs represented an important resource in lung transplantation. However, their use may be associated with a worse long-term graft and CLAD-free survival.

## Background

In lung transplantation (LTx), the imbalance between recipients and donors still remains an unresolved problem. Thus, the criteria for accepting a lung for transplantation have been increasingly expanded, and, by now, the “marginal” or extended-criteria donor (ECD) lungs form a fundamental organ pool.^1^, ^2^ Yet, some concerns have to be addressed.

A clear definition of ECD is lacking, as donor scores are based on reports made before on-site lung evaluation.^3^, ^4^, ^5^, ^6^, ^7^, ^8^ Additionally, many critical donor characteristics are excluded from these scores, and the long-term impact of ECD lungs on graft survival remains unexplored.^4^

At our institution, a high-volume Eurotransplant (ET) center, we have shown that ECD lungs can be safely used for stable, low-risk older recipients^9^, ^10^, ^11^ and for pediatric recipients,^12^ without any negative impact on short-term outcomes, and we have also assessed the impact of individual donor factors on graft function.^13^

Therefore, the aim of this retrospective study was to examine comprehensively the influence of donor lung quality, as defined by the Eurotransplant lung donor score (ET score), on long-term graft survival and function.

## Materials and methods

### Ethics statement

All patients signed a written informed consent regarding the use of their personal clinical data for research purposes at the time of listing for transplantation. In accordance with local German protocols, study approval by the institutional ethics review board was waived given the retrospective and noninterventional design of this study.

This study complies with the ethics statement of the International Society of Heart and Lung Transplantation (ISHLT).

### Study population

Records of adult and pediatric patients undergoing LTx at our Institution between January 2010 and May 2023 were retrospectively reviewed. All patients transplanted with a donor after brain death were included in the study and were divided into 3 groups, according to the corresponding donor ET score (ET score 6, group 1; ET scores 7 and 8, group 2; and ET scores 9-13, group 3). Patients receiving a living-donor lobar LTx or a combined heart and LTx were excluded. Follow-up ended on May 311, 2024 and was 100% completed.

### The Eurotransplant lung donor score

**Figure 1:**
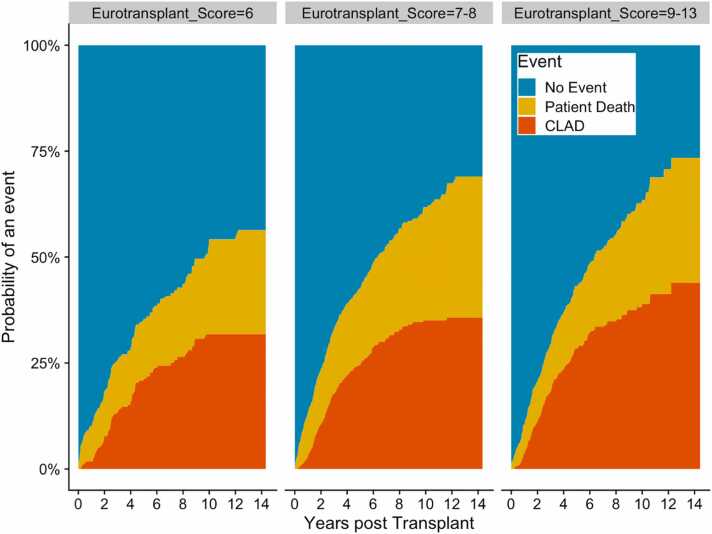
The competing-risk factor analysis for CLAD. The orange represents the patient fraction who died without having experienced CLAD. The height of the yellow stripe shows the fraction of patients who have experienced the named event, and the light blue shows the percentage of patients without the event at the time indicated at the x axis. CLAD, chronic lung allograft dysfunction.

The ET score, whose values span from 6 to 15, is a modification of the Oto's score and included donor age, pO_2_ at 100%FiO_2_ (mm Hg), smoking history, chest X-ray findings, bronchoscopy results, and donor medical history (malignancy, sepsis, drug abuse, meningitis, or positive virology hepatitis B surface antigen, hepatitis B core antibody, hepatitis C virus antibody) (Figure 1S, Supplemental Material).^4^ The ET score is usually not reported in the donor report but has to be retrospectively calculated.

In the present as well as in a previous study,^8^ donor lungs showing an ET score of 6 were considered ideal lungs, between 7 and 8 intermediate-risk lungs and between 9 and 15 ECD lungs. The ET score was used to quantify donor lung quality instead of other scores, as our institution is part of the ET network, which has adopted this standardized scoring system.

Table 1 shows the ET score distribution in the present study. No donors showed an ET score of 14 or 15.

**Table 1:** Donor Data

Variable	Group 1 (ET score 6, *n* = 280)	Group 2 (ET scores 7 and 8, *n* = 717)	Group 3 (ET scores 9-13, *n* = 506)	*p*-value
ET score				
6	280 (100)			
7		355 (49.5)		
8		362 (50.5)		
9			229 (45.3)	
10			151 (29.8)	
11			81 (16.0)	
12			37 (7.3)	
13			8 (1.6)	
14			0	
15			0	
Female sex	137 (48.9)	376 (52.4)	261 (51.6)	0.608
Age (years)	38 (22-48)	48 (35-57)	56 (41-63)	<0.001
<45	179 (63.9)	284 (39.6)	152 (30.0)	<0.001
45-54	101 (36.1)	197 (27.5)	80 (15.8)	<0.001
55-59	0	114 (15.9)	82 (16.2)	<0.001
≥60	0	122 (17.0)	192 (37.9)	<0.001
Age ≥70 years old	0	43 (6.0)	35 (6.9)	<0.001
Height (cm)	1.70 (1.65-1.80)	1.72 (1.65-1.80)	1.72 (1.67-1.80)	0.509
BMI (kg/m^2^)	24.2 (21.5-26.5)	25.0 (23.0-27.8)	26.0 (23.4-28.7)	<0.001
BSA (m^2^)	1.88 (1.70-2.00)	1.90 (1.80-2.00)	1.90 (1.80-2.10)	0.004
Donor-recipient size mismatch (cm)	2 (−2 to 9)	2 (−2 to 7)	2 (−3 to 7)	0.168
Ventilation time (days)	4 (3-7)	4 (3-7)	4 (3-8)	0.020
pO_2_ (100%, mm Hg)	439 (405-489)	404 (346-463)	327 (271-415)	<0.001
<300	0	68 (9.5)	187 (37.0)	<0.001
301-350	0	122 (17.0)	117 (23.1)	<0.001
351-450	159 (57.0)	316 (44.1)	119 (23.5)	<0.001
>450	120 (43.0)	211 (29.4)	83 (16.4)	<0.001
Smoking history	0	316 (44.1)	277 (54.7)	<0.001
Light^a^		96 (14.0)	79 (16.7)	
Medium^a^		108 (15.8)	104 (21.9)	
Strong^a^		79 (11.5)	60 (12.7)	
Drug abuse	0	0	50 (9.9)	<0.001
Alcohol abuse	45 (16.1)	261 (36.5)	180 (35.6)	<0.001
Lung contusion^b^	9 (3.2)	90 (12.6)	40 (7.9)	<0.001
Pneumonia	0	41 (5.7)	63 (12.5)	<0.001
Meningitis	0	0	29 (5.7)	<0.001
Sepsis	0	0	8 (1.6)	<0.001
Malignancy	0	0	23 (4.5)	<0.001
Hepatitis B (HBcAb+)	0	0	78 (15.4)	<0.001
Hepatitis C (HCVAb+)	0	0	6 (1.2)	0.003
Pulmonary arterial embolism	18 (6.4)	24 (3.4)	12 (2.4)	0.012
Bronchial asthma	2 (0.7)	11 (1.5)	3 (0.6)	0.235
Previous cardiac surgery	9 (3.2)	22 (3.1)	5 (1.0)	0.039
Diabetes mellitus, all types	9 (3.2)	48 (6.7)	51 (10.1)	0.001
Arterial hypertension	45 (16.1)	212 (29.6)	188 (37.2)	<0.001
Organ perfusion strategy				
EVLP	6 (2.1)	36 (5.0)	27 (5.3)	0.092
Celsior	248 (88.6)	611 (85.2)	439 (86.8)	0.363
Perfadex	22 (7.9)	67 (9.3)	37 (7.3)	0.423
Others^c^	4 (1.4)	3 (0.4)	3 (0.6)	0.205

Abbreviations: BAL, bronchoalveolar lavage; BSA, body surface area; BMI, body mass index; ET, Eurotransplant; EVLP, ex-vivo lung perfusion; HBcAb, hepatitis B core antibody; HCVAb, hepatitis C virus antibody.

Values are expressed as median (first to third quartiles) or N (%).

a The quantity (pack/years) was available for 526 out of 593 (89%) donors with a smoking history.

b The diagnosis of lung contusion was retrieved from the list of donor diagnoses present in the report.

c Other: Histidine and tryptophan a-ketoglutarate/Bretschneider, *n* = 9; University of Wisconsin, *n* = 1.

### Variable and outcome definitions

The primary outcome graft survival was defined as a composite end-point of patient survival and freedom from retransplantation. Lung graft function was evaluated at least 4 times during the first year after transplantation and subsequently 2 times per year, whenever possible, using lung function tests, chest imaging, as well as bronchoscopy with transbronchial biopsies to exclude infection, acute rejection, and obstructive airway complications (OAC). Acute rejection was defined either as the need for a pulsed-steroid therapy after exclusion of other causes of graft dysfunction (freedom from pulsed-steroid therapy) or according to the results of the transbronchial biopsies (freedom from biopsy-confirmed rejection).

Primary graft dysfunction (PGD) grade was defined according to the ISHLT guidelines^14^ and its calculation is reported as Supplemental Material. The definitions of chronic lung allograft dysfunction (CLAD) and OAC have been reported elsewhere.^15^, ^16^

### Donor management

Donor management at our institution has been previously reported.^9^, ^10^, ^11^, ^12^, ^13^ Briefly, donor after brain death lungs were perfused anterogradely and retrogradely with Celsior (Institut Georges Lopez Sas, Lissieu, France) solution as a standard pneumoplegia, and preserved en bloc in ice until the implantation of the first lung. Ex-vivo lung perfusion was not routinely used at our institution, but only during clinical studies which our institution had participated to.^17^, ^18^

Donor lungs were allocated by ET according to the recipient's lung allocating score (LAS). At our Institution, ECD lungs, offered by ET usually as a competitive or rescue allocation—meaning that such donor lungs had been previously refused by at least 4 other centers, mainly for poor quality—were preferably used for stable, low-risk older recipients^9^ and in some patients who, although on extracorporeal membrane oxygenation support (ECMO) or mechanical ventilation as a bridge to transplantation, had received a lower LAS due to their underlying lung disease, and whose clinical conditions did not allow waiting for a more ideal donor.

### Donor characteristics

Table 1 reports the donor characteristics. ECDs (group 3, ET scores 9-13) were older (*p* < 0.001), showed more often an impaired oxygenation capacity (*p* < 0.001), an active or past smoking and drug abuse history (*p* < 0.001 in both cases), a radiologic and bronchoscopic evidence of pneumonia (*p* < 0.001), a history of meningitis (*p* < 0.001), sepsis (*p* < 0.001), hepatitis B and C (*p* < 0.001 and *p* = 0.003, respectively), and a past history of malignancy (*p* < 0.001), than the other 2 donor groups. Interestingly, group 1 donors showed an acute pulmonary arterial embolism and had previously undergone a cardiac surgical procedure more often than group 2 and 3 donors (6.4% vs 3.4% vs 2.4%, *p* = 0.012; 3.2% vs 3.1% vs 1%, *p* = 0.039, respectively).

### Recipient management

Recipient management at our institution has been described in previous publications.^9^, ^10^, ^11^, ^12^, ^13^, ^19^, ^20^ ECMO was preferred over cardiopulmonary bypass (CPB) for intraoperative and postoperative support.^19^ Patients did not receive any induction therapy and underwent triple immunosuppressive therapy including a calcineurin inhibitor (primarily Tacrolimus since 2013), mycophenolate mofetil, and prednisolone. At follow-up, mycophenolate mofetil was switched to Everolimus or Azathioprine in some patients.

### Data analysis

SPSS 28.0 (IBM, New York, NY) and R (R Foundation for Statistical Computing, Vienna, Austria) were used for data analysis.

Primary end-point was graft survival, defined as a composite end-point of patient survival and freedom from retransplantation. Secondary end-points were PGD grade 3 at 72 hours after transplantation, CLAD, acute rejection, and OACs.

Categorical and continuous variables were summarized as percentages and median (interquartile range), and compared between groups using the chi-square and nonparametric Kruskal-Wallis tests, respectively. Survival and freedom from end-points were calculated by the product-limit method of Kaplan-Meier, reported as % and 95% confidence interval (CI), and compared between groups using the log-rank test. Given the competing risks for nonfatal events (CLAD) and death within the cohort, further analysis to define the rates of patients dying with or without a previous nonfatal event was performed.

The variables included in the multivariable analyses were reported in Table 1, Table 2, Table 3. The risk factor analysis for PGD grade 3 at 72 was performed using a binary logistic regression analysis. Goodness-of-fit was tested using the Hosmer-Lemeshow test. The risk factor analysis for time-dependent outcomes was performed using the Cox's multivariable analysis. The proportional hazards assumption was tested by including the time-dependent coefficients in the regression models. Factors, that did not satisfy this assumption were not included in the final multivariable model.

**Table 2:** Preoperative Recipient Data

Variable	Group 1 (ET score 6, *n* = 280)	Group 2 (ET scores 7 and 8, *n* = 717)	Group 3 (ET scores 9-13, *n* = 506)	*p*-value
Female sex	120 (42.9)	346 (48.3)	240 (47.4)	0.298
Age (years)	44 (22-56)	53 (40-59)	55 (45-60)	<0.001
Pediatric patients (<18 years old)	55 (19.6)	37 (5.2)	20 (4.0)	<0.001
Age >60 years old	30 (10.7)	124 (17.3)	120 (23.7)	<0.001
Weight (kg)	62 (48-77)	66 (55-78)	67 (55-78)	<0.001
Height (cm)	169 (160-178)	170 (163-178)	171 (164-178)	0.071
BSA (m^2^)	1.70 (1.48-1.90)	1.77 (1.59-1.95)	1.77 (1.61-1.92)	0.002
BMI (kg/m^2^)	21.0 (17.4-25.2)	22.7 (19.4-25.6)	22.8 (19.6-25.9)	<0.001
CMV risk profile				
No risk (D^−^/R^−^)	61 (21.9)	147 (20.5)	90 (17.8)	0.332
Intermediate risk (D^−^/R^+^, D^+^/R^+^)	133 (47.7)	353 (49.2)	270 (53.5)	0.210
High risk (D^+^/R^−^)	85 (30.5)	217 (30.3)	145 (28.7)	0.811
Previous thoracic operations	66 (23.6)	150 (20.9)	89 (17.6)	0.115
Severe coronary artery disease	23 (8.2)	62 (8.6)	49 (9.7)	0.740
Previous smoking history	54 (19.3)	284 (39.6)	217 (42.9)	<0.001
Transplant indication				
COPD	41 (14.6)	209 (29.1)	193 (38.1)	<0.001
Pulmonary fibrosis	86 (30.7)	247 (34.4)	162 (32.0)	0.458
Cystic fibrosis	74 (26.4)	115 (16.0)	68 (13.4)	<0.001
Pulmonary arterial hypertension	23 (8.2)	55 (7.7)	22 (4.3)	0.036
Sarcoidosis	7 (2.5)	23 (3.2)	18 (3.6)	0.722
Redo transplantation	28 (10.0)	41 (5.7)	21 (4.2)	0.004
Others^a^	21 (7.5)	27 (3.8)	21 (4.2)	0.034
LAS^b^	37.8 (33.9-46.9)	35.9 (32.6-42.6)	34.8 (32.6-40.7)	<0.001
Cytotoxic antibodies				
Anti-HLA I	41 (14.6)	112 (15.6)	82 (16.2)	0.846
Anti-HLA II	61 (21.8)	135 (18.8)	100 (19.8)	0.572
Waiting list time (days)	52 (17-153)	53 (14-177)	43 (14-138)	0.192
Mechanical ventilation	12 (4.3)	21 (2.9)	21 (4.2)	0.416
Intensive care unit	31 (11.1)	78 (10.9)	45 (8.9)	0.466
ECMO as a bridge to transplantation	20 (7.1)	51 (7.1)	36 (7.1)	1.000
Time on ECMO preoperatively (days)	13 (7-28)	9 (5-30)	10 (5-19)	0.497

Abbreviations: BMI, body mass index; BSA, body surface area; CMV, cytomegalovirus; COPD, chronic obstructive pulmonary disease; D, donor; ECMO, extracorporeal membrane oxygenation; ET, Eurotransplant; HLA, human leukocyte antigen; LAS, lung allocation score; R, recipient.

Values are expressed as median (first to third quartiles) or N (%).

a Among them, graft vs host disease involving the lung (GvHD, *n* = 17); lung involvement in patients with Histiocytosis X (*n* = 6); acute respiratory distress syndrome/post-COVID19 (*n* = 10); bronchiectasis (*n* = 22).

b LAS available from January 2012 (*n* = 1,259).

**Table 3:** Intraoperative and postoperative course, before hospital discharge.

Variable	Group 1 (ET score 6, *n* = 280)	Group 2 (ET scores 7 and 8, *n* = 717)	Group 3 (ET scores 9-13, *n* = 506)	*p*-value
*Intraoperative characteristics*				
Thoracotomy				
Sternum sparing thoracotomies	250 (89.3)	657 (91.6)	465 (91.9)	0.416
Clamshell	29 (10.4)	60 (8.4)	41 (8.1)	0.523
Double lung	271 (96.8)	694 (96.8)	499 (98.6)	0.109
Ischemic time (minutes)				
First lung	394 (309-492)	390 (321-497)	386 (312-467)	0.372
Second lung	516 (438-621)	524 (439-630)	517 (440-604)	0.224
Intraoperative support				
CPB	12 (4.3)	16 (2.2)	16 (3.2)	0.208
ECMO	82 (29.3)	217 (30.3)	157 (31.0)	0.877
Concomitant CABG	5 (1.8)	11 (1.5)	9 (1.8)	0.932
Combined lung and liver transplantation	5 (1.8)	4 (0.6%)	3 (0.6%)	0.120
Blood products, intraoperative				
PRBCs (units)	2 (0-4)	2 (0-4)	2 (0-4)	0.421
PC (units)	0 (0-2)	0 (0-2)	0 (0-2)	0.906
FFP (units)	4 (2-6)	4 (2-6)	4 (3-6)	0.264
Postoperatively extended ECMO support	32 (11.4)	77 (10.7)	51 (10.1)	0.836
Lung volume reduction surgery				
Atypical resection	3 (1.1)	11 (1.5)	13 (2.6)	0.243
Lobar resection	10 (3.6)	22 (3.1)	14 (2.8)	0.821
*Postoperative characteristics*				
PGD score grade 3				
At 24 hours	16 (5.8)	45 (6.3)	30 (5.9)	0.943
At 48 hours	16 (5.8)	42 (5.9)	31 (6.2)	0.969
At 72 hours	11 (4.0)	36 (5.0)	28 (5.5)	0.627
Rethoracotomy for bleeding	20 (7.1)	69 (9.6)	42 (8.3)	0.425
Blood products, overall				
PRBCs (units)	6 (3-12)	6 (4-12)	6 (4-13)	0.462
PC (units)	1 (0-2)	1 (0-2)	1 (0-2)	0.955
FFP (units)	5 (4-8)	5 (4-9)	5 (4-9)	0.852
Dialysis				
New	21 (7.5)	72 (10.0)	37 (7.3)	0.188
At discharge^a^	10 (3.6)	40 (5.6)	25 (5.0)	0.425
Early donor-specific antibodies				
Anti-HLA I	16 (5.7)	37 (5.2)	37 (7.3)	0.284
Anti-HLA II	63 (22.5)	140 (19.5)	109 (21.6)	0.500
Pulsed-steroid therapy	72 (25.8)	202 (28.2)	143 (28.3)	0.712
Wound healing disorders	14 (5.0)	52 (7.3)	30 (5.9)	0.374
Secondary ECMO therapy	3 (1.1)	21 (2.9)	11 (2.2)	0.209
Tracheostomy	27 (9.6)	91 (12.7)	61 (12.1)	0.407
Ventilation time (hours)	12 (8-26)	14 (9-37)	13 (9-30)	0.062
ICU stay (days)	2 (1-5)	2 (1-5)	2 (1-5)	0.090
Hospital stay (days)	24 (21-33)	24 (21-34)	24 (21-33)	0.969

Abbreviations: CABG, coronary artery bypass grafting; CPB, cardiopulmonary bypass; ECMO, extracorporeal membrane oxygenation; ET, Eurotransplant; FFP, fresh frozen plasma; HLA, human leukocyte antigen; ICU, intensive care unit; PC, platelet concentrate; PGD, primary graft dysfunction; PRBCs, packed red blood cells.

Values are expressed as median (first to third quartiles) or N (%).

a In-hospital deaths included.

*p*-Values ≤0.05 were considered statistically significant.

## Results

### Patient groups

Between January 2010 and May 2023, 1,538 patients underwent LTx at our institution. Three (0.2%) patients undergoing living-donor lobar LTx and 32 (2%) patients undergoing combined heart and LTx were excluded. Among the included 1,503 (97.8%) recipients, 280 (19%) patients were transplanted with a lung showing an ET score of 6 (group 1), 717 (48%) patients with a lung showing an ET score between 7 and 8 (group 2), and 506 (34%) patients with a lung showing an ET score between 9 and 13 (ECD lung recipients, group 3).

### Pretransplant recipient characteristics

Table 2 reports pretransplant recipient characteristics. Median recipient age differed significantly between groups (*p* < 0.001), being 55 (45-60) years in group 3 recipients and 44 (22-56) years in group 1 recipients. Similarly, transplant indications were differently distributed between groups. Group 3 recipients were transplanted more often for chronic obstructive pulmonary disease (COPD) and group 1 recipients for cystic fibrosis, primary pulmonary hypertension, or underwent redo transplantation. The need for pretransplant mechanical ventilation or ECMO as a bridge to transplantation was similar in all 3 groups (*p* = 0.416 and 1.000, respectively). Group 1 recipients showed a higher LAS (*p* < 0.001).

### Intraoperative and postoperative course

Table 3 reports the intraoperative and postoperative course. The intraoperative use of ECMO or CPB did not differ between groups (*p* = 0.877 and 0.208, respectively).

The prevalence of major post-transplant complications as well as ventilation, intensive care unit, and hospital stay times did not differ between groups (Table 3). In-hospital mortality did not differ between groups (*p* = 0.713, Table 4).

**Table 4:** Outcomes

Variable	Group 1 (ET score 6, *n* = 280)	Group 2 (ET scores 7 and 8, *n* = 717)	Group 3 (ET scores 9 and 13, *n* = 506)	*p*-value
Graft survival (years)				
1	91 (87, 95)	89 (87, 91)	91 (89, 93)	
5	74 (68, 80)	68 (64, 72)	70 (66, 74)	
7	69 (63, 75)	60 (56, 64)	61 (55, 67)	
10	56 (48, 64)	51 (47, 55)	48 (42, 54)	0.052
Patient survival, overall (years)				
1	91 (87, 95)	89 (87, 91)	92 (90, 94)	
5	78 (72, 84)	71 (67, 75)	72 (68, 76)	
10	61 (53, 69)	53 (49, 57)	50 (44, 56)	0.011
Patient survival conditioned to hospital discharge (years)				
1	96 (94, 98)	94 (92, 96)	96 (94, 98)	
5	82 (76, 88)	74 (70, 78)	75 (71, 79)	
10	64 (56, 72)	56 (52, 60)	53 (47, 59)	0.007
In-hospital mortality	13 (4.6)	37 (5.2)	21 (4.2)	0.713
Infection	5 (38.5)	19 (51.4)	10 (47.6)	
Graft dysfunction	3 (23.1)	8 (21.6)	4 (19.0)	
Cardiac	1 (7.7)	4 (10.8)	5 (23.8)	
Bleeding	1 (7.7)	2 (5.4)	0	
Malignancy	0	0	1 (4.8)	
Cerebrovascular event	2 (15.4)	3 (8.1)	1 (4.8)	
Other	1 (7.7)	1 (2.7)	0	
Causes of death after hospital discharge				
CLAD	26 (9.7)	77 (11.3)	63 (12.9)	0.404
Infection	16 (6.0)	80 (11.7)	45 (9.2)	0.024
Malignancy	11 (4.1)	27 (4.0)	16 (3.3)	0.788
Cardiac	6 (2.2)	23 (3.4)	14 (2.9)	0.644
Other	9 (3.4)	43 (6.3)	25 (5.1)	0.187
Freedom from CLAD^a^ (years)	(*n* = 262)	(*n* = 669)	(*n* = 478)	
3	85 (81, 89)	80 (76, 84)	80 (76, 84)	
5	76 (70, 82)	71 (67, 74)	68 (64, 72)	
7	72 (66, 78)	63 (59, 67)	61 (55, 67)	
10	63 (55, 71)	57 (51, 63)	55 (49, 61)	0.033
Freedom from redo transplantation (years)				
1	99 (98, 100)	99 (98, 100)	99 (98, 100)	
5	95 (92, 98)	96 (94, 98)	98 (97, 99)	
10	90 (84, 96)	95 (93, 97)	94 (90, 98)	0.266

Abbreviations: CLAD, chronic lung allograft dysfunction; ET, Eurotransplant.

Values are expressed as mean % (95% confidence interval) for survival results, median (interquartile range), or N (%).

a About 1,409 (94%) patients were considered for CLAD analysis.

Prevalence of PGD grade 3 at 72 hours after transplantation did not differ between groups (4% vs 5% vs 5.5%, in groups 1, 2 and 3 patients, respectively, *p* = 0.425). At the multivariable analysis (Table 1S, Supplemental Material), among donor characteristics, only donor age (years) was significantly associated with PGD grade 3 at 72 hours (odds ratio (OR) = 1.030, 95%CI = 1.030-1.048, *p* = 0.040).

### Primary outcome

Median follow-up was 64 (30-104) months. Graft survival was worse in group 2 (ET scores 7 and 8) and 3 (ET scores 9-13) recipients than in group 1 (ET score 6) recipients (*p* = 0.052). This difference became evident after 5 years follow-up, amounting the 10-year graft survival to 56%, 51%, and 48% in group 1 vs 2 vs 3 recipients, respectively (Table 4, Figure 1S-A, Supplemental Material). A similar trend was observed for overall patient survival and survival conditioned to hospital discharge (*p* = 0.011 and 0.007, respectively, Table 4).

At the Cox's analysis (Table 5), the ET score itself as well as transplantation using an ECD (ET scores 9-13) lung was not significantly associated with worse graft survival (hazard ratio (HR) = 1.029, 95%CI = 0.979-1.081, *p* = 0.257; HR = 1.038, 95%CI = 0.876-1.231, *p* = 0.666, respectively). However, among the single donor characteristics included in the ET score and other donor characteristics reported in Table 2, smoking history was the only variable significantly associated with worse recipient graft survival (HR = 1.260, 95%CI = 1.072-1.481, *p* = 0.005). More recipient than donor characteristics were associated with graft survival (Table 5).

**Table 5:** Cox Multivariable Analysis for Graft Survival

Variable	Univariable				Multivariable		
Mortality or retransplant (*n* = 561)	HR	95%CI	*p*-value	*p*-value TCOV*variable^a^	HR	95%CI	*p*-value
*Categorical variables*							
Pediatric patients	0.651	0.456-0.929	0.018	0.075			
Recipient age >60 years old^a^	1.453	1.192-1.771	<0.001	0.002			
Previous thoracic operations	1.212	0.994-1.478	0.058	0.076			
Severe CAD	1.549	1.199-2.001	<0.001	0.232	1.439	1.106-1.872	0.007
Previous smoking history	1.177	0.998-1.388	0.053	0.065			
CMV risk profile, low risk	0.814	0.662-1.000	0.050	0.564	0.805	0.654-0.990	0.040
Pulmonary fibrosis	1.279	1.083-1.510	0.004	0.519	1.222	1.030-1.449	0.021
Cystic fibrosis^a^	0.596	0.473-0.753	<0.001	0.005			
Double-lung transplantation	0.435	0.291-0.649	<0.001	0.310			
Single-lung transplantation	2.300	1.540-3.434	<0.001	0.310			
Thoracotomy, sternum sparing^a^	0.757	0.574-0.999	0.050	0.034			
Thoracotomy, clamshell^a^	1.312	0.992-1.736	0.057	0.021			
Intraoperative support, ECMO^a^	1.191	1.001-1.417	0.048	<0.001			
Lung volume reduction surgery	1.616	1.177-2.220	0.003	0.231			
PGD score, grade 3, 24 hours^a^	1.634	1.216-2.195	0.001	0.011			
PGD score, grade 3, 48 hours^a^	1.514	1.110-2.065	0.009	0.002			
PGD score, grade 3, 72 hours^a^	2.197	1.605-3.008	<0.001	<0.001			
Rethoracotomy for bleeding^a^	1.964	1.538-2.509	<0.001	<0.001			
Dialysis, new^a^	2.944	2.342-3.700	<0.001	<0.001			
Pulsed-steroid therapy	1.409	1.190-1.670	<0.001	0.253	1.196	1.000-1.429	0.049
Wound healing disorder^a^	1.460	1.090-1.956	0.011	0.024			
Secondary ECMO therapy^a^	4.280	2.908-6.300	<0.001	0.003			
Tracheostomy^a^	2.223	1.806-2.736	<0.001	<0.001			
IgGAM therapy for eDSA	0.814	0.646-1.026	0.081	0.068			
Cyclosporine at discharge^a^	1.499	1.256-1.788	<0.001	0.020			
Tacrolimus at discharge^a^	0.667	0.559-0.796	<0.001	0.032			
Donor, ET score 6	0.765	0.615-0.951	0.016	0.309			
Donor age <45 years old^a^	0.800	0.678-0.945	0.008	<0.001			
Donor, smoking history	1.213	1.033-1.425	0.018	0.283	1.260	1.072-1.481	0.005
Donor, alcohol abuse	1.174	0.996-1.383	0.056	0.784			
Donor, meningitis	0.322	0.134-0.777	0.012	0.660	0.345	0.143-0.834	0.018
Donor, pO_2_ 301-350 mm Hg	1.222	0.993-1.504	0.058	0.945			
Donor, pO_2_ 351-450 mm Hg	0.852	0.721-1.006	0.060	0.435			
*Continuous variables*							
Recipient age (years)^a^	1.015	1.009-1.021	<0.001	<0.001			
Recipient BMI (kg/m^2^)^a^	1.040	1.021-1.060	<0.001	0.014			
Ischemic time, second lung (minutes)	1.001	1.000-1.001	0.010	0.299			
Blood products, PRBCs (units)^a^	1.016	1.013-1.019	<0.001	<0.001			
Blood products, PC (units)^a^	1.043	1.033-1.052	<0.001	<0.001			
Blood products, FFPs (units)^a^	1.013	1.009-1.017	<0.001	0.002			
Ventilation time (hours)	1.015	1.012-1.017	<0.001	0.286			
ICU stay (days)^a^	1.016	1.013-1.020	<0.001	0.023			
Hospital stay (days)	1.007	1.005-1.009	<0.001	0.255	1.007	1.005-1.008	<0.001
Donor age (years)^a^	1.007	1.002-1.012	0.003	<0.001			

Abbreviations: BMI, body mass index; CAD, coronary artery disease; CI, confidence interval; CPB, cardiopulmonary bypass; CMV, cytomegalovirus; ECMO, extracorporeal membrane oxygenation; eDSA, early anti-HLA donor-specific antibodies; ET, Eurotransplant; FFP, fresh frozen plasma; HLA, human leukocyte antigen; ICU, intensive care unit; PC, platelet concentrate; PGD, primary graft dysfunction; PRBCs, packed red blood cells; TCOV, time-dependent covariate.

a These variables did not satisfy the Cox proportional hazard assumption (*p*-value TCOV*variable >0.05) and were not included in the final multivariable analysis.

### Secondary outcomes and graft function

Forced expiratory volume in 1 sec (% predicted) measured at discharge, at 1-year follow-up, and at last available outpatient control as well as the acute-rejection-free survival (defined as freedom from pulsed-steroid therapy for presumed rejection and freedom from biopsy-confirmed rejection) did not differ between groups (Table 6). OAC-free survival was worse in groups 1 and 3 recipients than in group 2 recipients (*p* = 0.024).

**Table 6:** Outcomes (Continued)

Variable	Group 1 (ET score 6, *n* = 280)	Group 2 (ET scores 7 and 8, *n* = 717)	Group 3 (ET scores 9-13, *n* = 506)	*p*-value
Freedom from biopsy-confirmed rejection^a^	(*n* = 233)	(*n* = 624)	(*n* = 451)	
6 months	71 (65, 77)	68 (64, 72)	67 (63, 71)	
1 year	65 (59, 71)	64 (60, 68)	61 (57, 65)	
3 years	58 (52, 64)	54 (50, 58)	53 (49, 57)	0.467
Number of biopsies per patient	4 (3-6)	4 (3-6)	5 (3-6)	0.274
ISHLT grade A ≥1^b^				
A1	88 (37.5)	245 (39.3)	175 (38.8)	0.917
A2	33 (14.2)	80 (12.8)	74 (16.4)	0.257
A3	5 (2.1)	3 (0.5)	5 (1.1)	0.088
A4	0	0	0	
Freedom from pulsed-steroid therapy^c^	(*n* = 267)	(*n* = 680)	(*n* = 483)	
6 months	67 (61, 73)	66 (62, 70)	67 (63, 71)	
1 year	61 (55, 67)	60 (56, 64)	60 (56, 64)	
3 years	50 (44, 56)	47 (43, 51)	46 (42, 50)	0.666
Number of pulsed-steroid therapies	1 (0-1)	1 (0-1)	1 (0-2)	0.840
Freedom from infection	(*n* = 267)	(*n* = 680)	(*n* = 484)	
1 year	75 (69, 81)	74 (70, 78)	76 (72, 80)	
5 years	49 (43, 55)	46 (42, 50)	47 (42, 52)	
10 years	27 (19, 35)	28 (24, 32)	24 (18, 30)	0.913
Number of admissions for infections	1 (0-2)	1 (0-2)	1 (0-1)	0.532
Freedom from obstructive airway complications	(*n* = 271)	(*n* = 698)	(*n* = 491)	
3 months	91 (87, 95)	94 (92, 96)	92 (90, 94)	
1 year	84 (80, 88)	89 (87, 91)	84 (80, 88)	
5 years	84 (80, 88)	89 (87, 91)	84 (80, 88)	0.024
Immunosuppressive therapy at discharge				
Tacrolimus	232 (82.9)	543 (75.7)	432 (85.4)	<0.001
Cyclosporine	48 (17.1)	176 (24.5)	76 (15.0)	<0.001
Immunosuppressive therapy at last control	(*n* = 267)	(*n* = 678)	(*n* = 482)	
Tacrolimus	227 (85.0)	553 (81.6)	396 (82.2)	0.447
Cyclosporine	38 (14.2)	118 (17.4)	83 (17.2)	0.473
Everolimus	42 (15.7)	86 (12.7)	73 (15.1)	0.342
Mycophenolate mofetil	218 (81.6)	550 (81.1)	374 (77.6)	0.255
IgGAM therapy for eDSA	58 (20.7)	117 (16.3)	100 (19.8)	0.154
FEV_1_ (% predicted)				
Pretransplant	25 (18-42)	27 (18-44)	25 (18-40)	0.213
At discharge	66 (55-80)	66 (53-81)	67 (55-80)	0.789
At 1 year after transplantation	89 (72-104)	86 (70-103)	85 (68-102)	0.233
At last control	77 (53-96)	74 (48-95)	73 (50-94)	0.385

Abbreviations: eDSA, early anti-HLA donor-specific antibodies; ET, Eurotransplant; FEV1, forced expiratory volume in 1 sec; HLA, human leukocyte antigen; IgGAM, intravenous human IgA- and IgM-enriched immunoglobulins.

Values are expressed as mean % (95% confidence interval) or median (first to third quartiles).

a Biopsies were available from 1,308 (87%) patients.

b Patients with at least 1 biopsy with a grade A ≥1 are reported.

c This end-point does not include the pulsed-steroid therapies performed during the initial hospitalization after lung transplantation (Table 3).

CLAD-free survival (%) was worse in group 2 and 3 recipients than in group 1 recipients (at 5 years, 71 and 68 vs 76; at 10 years, 57 and 55 vs 63, respectively, *p* = 0.033, Table 4 and Figure 1S-B, Supplemental Material). The competing-risk analysis showed a significant difference in CLAD incidence only between group 1 and 3 recipients (*p* = 0.016, Figure 1). At the Cox's analysis (Table 7), transplantation using an ECD (ET scores 9-13) lung did not emerge as a risk factor for worse CLAD-free survival (HR = 1.183, 95%CI = 0.973-1.440, *p* = 0.093). Smoking history was the only donor characteristic significantly associated with worse CLAD-free survival (HR = 1.466, 95%CI = 1.215-1.769, *p* < 0.001).

**Table 7:** Cox Multivariable Analysis for Chronic Lung Allograft Dysfunction (CLAD)

Variable	Univariable				Multivariable		
CLAD (*n* = 437)	HR	95%CI	*p*-value	*p*-value TCOV*variable^a^	HR	95%CI	*p*-value
*Categorical variables*							
Previous thoracic operations	0.741	0.567-0.968	0.028	0.589			
CMV risk profile, high risk	1.301	1.071-1.581	0.008	0.194			
CMV risk profile, low risk	0.730	0.569-0.937	0.013	0.481	0.732	0.571-0.939	0.014
Transplant indication, COPD	1.189	0.972-1.455	0.093	0.288			
Intraoperative support, CPB	0.355	0.133-0.950	0.039	0.550			
Lung volume reduction surgery	1.965	1.356-2.847	<0.001	0.927	1.915	1.322-2.776	<0.001
Dialysis, new	0.607	0.356-1.033	0.066	0.943			
eDSA treatment, IgGAM^a^	0.750	0.574-0.979	0.035	0.031			
Cyclosporine at discharge^a^	2.137	1.751-2.608	<0.001	0.007			
Tacrolimus at discharge^a^	0.465	0.381-0.567	<0.001	0.010			
Donor, ET score 6	0.726	0.561-0.939	0.015	0.745			
Donor, ET scores 9-13	1.183	0.973-1.440	0.093	0.492			
Donor, smoking history	1.469	1.218-1.773	<0.001	0.775	1.466	1.215-1.769	<0.001
*Continuous variables*							
Donor, Eurotransplant score	1.073	1.014-1.136	0.015	0.181			

Abbreviations: COPD, chronic obstructive pulmonary disease; CPB, cardiopulmonary bypass; CMV, cytomegalovirus; eDSA, early anti-HLA donor-specific antibodies; ET, Eurotransplant; HLA, human leukocyte antigen; IgGAM, intravenous IgA- and IgM-enriched human immunoglobulins; TCOV, time-dependent covariate.

a These variables did not satisfy the Cox proportional hazard assumption (*p*-value TCOV*variable >0.05) and were not included in the final multivariable analysis.

### Outcome stratification in group 3 (ET scores 9-13) recipients

Outcomes in group 3 patients were further stratified according to the availability of a donor thoracic computed tomography (CT) before retrieval and donor age ≤55 years (Table 2, Table 3, respectively, Supplemental Material). In the former analysis, freedom from CLAD was worse in patients without any CT before retrieval (*p* = 0.044). In the latter analysis, a better trend in patient and graft survival was observed in group 3 recipients of donors >55 years (*p* = 0.060 and *p* = 0.069).

## Discussion

This single-center retrospective study showed that ideal (ET score 6) lung donors represented only 19% of our donor pool, while more than 30% of lung donors were classified as extended-criteria (ET scores 9-13) donors. ECD lungs represented an important organ pool, not only for older patients transplanted for COPD but also for those recipients on ECMO or mechanical ventilation as a bridge to transplantation. The use of ECD lungs did not impair the early post-transplant course, with a similar prevalence of PGD grade 3 between groups, and did not impair the short- and mid-term graft survival and function. However, long-term graft survival and CLAD-free survival were worse in recipients of nonideal donors, particularly ECD donors. At the multivariable analysis, among donor characteristics, only smoking history was associated with worse graft survival and CLAD-free survival. The ET score itself and other donor characteristics not included in the score, such as acute pulmonary embolism, history of asthma, and previous cardiac surgical operations among others, were not associated with worse outcomes.

Our study, while confirming the short-term survival results previously reported by a multicentric study using the ET score,^4^ presented comparatively a longer follow-up time and evaluated additional outcomes such as PGD and CLAD.

The negative impact of ECD lungs on long-term graft survival and CLAD-free survival should not discourage their use in transplantation for the following reasons. First, survival and CLAD-free survival in our ECD (ET scores 9-13, group 3) recipients were better than those reported by the 36th ISHLT adult lung and heart-LTx report (1-, 5-, 10-year survival rates: 81%, 56%, and 34%; 3-, 5-, 10-year bronchiolitis obliterans syndrome-free survival: 67%, 50%, and 23%, respectively).^21^ Moreover, the life expectancy, for both recipients with COPD^22^ and recipients on ECMO or mechanical ventilation as a bridge to transplantation, would have been lower without transplantation. Several studies have investigated the use of ECD lungs and their impact on short-term survival. Christie et al comprehensively examined all organs transplanted in the United States between 2005 and 2018 and did not reveal any difference in survival rates at 30 days, 90 days, and 1 year.^23^ Halpern et al reported comparable findings and did not observe any notable adverse short-term outcomes in institutions that adopted an especially proactive approach in utilizing nonideal donor organs.^24^

In addition, the number of ECD donors is expected to further increase in the next future. In ET area, median donor age has increased from almost 40 years in 2007 to 50 years in 2023.^25^ Similarly, in Germany, the percentage of lung donors older than 75 years has increased from 0% in 2007 to 10% in 2023.^26^

In our study, many donor characteristics used to define a lung as marginal were not associated with worse outcomes at follow-up. The results confirmed those previously published by our group^13^ and were also in line with the results published by other groups regarding the impact of post-traumatic contusion, substance abuse, presence of previous cardiac surgical procedures.^27^, ^28^, ^29^, ^30^, ^31^ However, increasing donor age was associated with the development of PGD grade 3 at 72 hours after transplantation (Table 1S).

The association between a positive donor smoking history and worse long-term outcomes points out a structural irreversible pulmonary damage provoked by smoking. The smoking-induced activity of matrix metalloproteinases degrading extracellular matrix components such as collagen and elastin may lead to tissue remodeling and fibrosis, reducing lung elasticity and resulting in increased susceptibility to fibrosis and chronic rejection*.*^32^ However, rejecting organs from these donors is not acceptable, because this decision would increase the recipient waiting list time and mortality. Rather, it would be advisable to perform a chest CT scan for these donors to exclude the presence of pulmonary emphysema and malignancy. The lower CLAD incidence in group 3 patients receiving a CT before retrieval supports this strategy. Other studies have shown a negative impact of donor smoking history on short- and long-term survival.^33^, ^34^, ^35^ In particular, Bonser et al reported higher mortality at 1, 3, and 5 years in patients receiving organs from smokers. Moreover, they evaluated the impact of accepting donor organs with a smoking history vs remaining on the waiting list and showed that survival was better when a donor organ with smoking history was accepted than remaining on the waiting list and waiting for a donor organ without a smoking history.^36^

Our study showed that recipient characteristics, such as presence of significant coronary artery disease and pulmonary fibrosis, might have a major impact on graft survival than donor characteristics. Recipient age may have confounded the impact of ECD lungs on survival, as ECD lungs were often allocated to older COPD and fibrosis patients at our center, possibly explaining survival differences.^9^, ^10^, ^11^ Kaplan-Meier curves (Figure 2S-A) show that patient and graft survival differences became evident only at long term. However, recipient age was excluded from the multivariable model, because it did not satisfy the Cox proportional hazard assumption. The significant role of recipient characteristics in influencing results after transplantation with ECD lungs has been underlined by other authors.^37^, ^38^, ^39^ Among others, Ehrsam et al showed a significantly shorter median survival in older patients. Recipient age was a significant risk factor for mortality in the univariable but not in the multivariable analysis. The authors emphasized that recipient comorbidity profile exerted a more substantial influence on long-term survival.^39^ These results underline the need for establishing a donor-recipient matchmaker in addition to the available allocation tools, to optimize organ utilization.

## Study limitations

A limitation of this study lies in the difficulty in defining the marginality of a donor lung, because donor scores are based on preretrieval donor information, potentially misclassifying some functionally ideal lungs as marginal. Propensity score matching between recipient groups was considered but limited by donor-recipient heterogeneity and reduced sample size. Therefore, multivariable Cox regression was employed for a more robust analysis, adjusting for confounders. Additionally, our allocation strategy, pairing younger recipients with younger donors and older recipients with older donors, might have introduced a selection bias, affecting graft survival outcomes.

## Conclusions

ECD lungs constituted one-third of our donor pool and represented an important resource in the current era of donor organ shortage. The negative impact of ECD donors on long-term graft survival and CLAD-free survival should not discourage their use in LTx.

## Author contributions

All authors have contributed to preparing and drafting the manuscript, and no person or persons other than the authors listed have contributed significantly to its preparation. In particular, research was designed by K.F., N.D.M., and F.I.; research was conducted by K.F., F.I., N.D.M., and S.K.; data analysis was performed by K.F., S.K., and F.I.; writing and revision of the manuscript were performed by K.F., N.D.M., and F.I.; and critical revision of the manuscript was performed by K.A., F.W., D.B., K.M., J.C., N.S., M.A., A.R., C.K.; J.S., and F.I. share senior authorship; K.F. and N.D.M. share first authorship. All authors have read and approved the manuscript as written.

## Disclosure statement

Fabio Ius declares a consulting contract with Biotest AG and congress travel grants paid by Abbott, outside the submitted work. Sophie Kruszona, Khalil Aburahma, Philipp Wand declare congress travel grants paid by Biotest AG, Xvivo, and Abbott, outside the submitted work. The remaining authors do not declare any conflict of interest.

Acknowledgements and Funding: None.

## Footnotes

Appendix A Supplementary data associated with this article can be found in the online version at doi:10.1016/j.jhlto.2024.100166.
