# Supplementary material for: Impact of donor organ quality on recipient outcomes in lung transplantation: 14-Year single-center experience using the Eurotransplant lung donor score
Source: JHLT Open. 2024 Oct 11;6:100166. doi: 10.1016/j.jhlto.2024.100166 (PMC11935426; doi:10.1016/j.jhlto.2024.100166)
Supplement: Supplementary file 1 — Supplementary material. [file mmc1.docx]

**SUPPLEMENTAL MATERIAL**

**Impact of donor quality on recipient outcomes in lung transplantation: 14-year single-center experience using the Eurotransplant lung donor score**

Katharina Flöthmann^1,5^, Nunzio Davide de Manna MD^1,5^, Khalil Aburahma MD^1^, Sophie Kruszona^1^, Philipp Wand MD^1^, Dmitry Bobylev MD^1^, Carsten Müller MD^2^, Julia Carlens MD^2^, Nicolaus Schwerk MD^2^, Murat Avsar MD^1^, Arjang Ruhparwar MD^1,4^, Christian Kühn MD^1,4^, Mark Greer MD^3,4^, Jawad Salman MD^1,6^, Fabio Ius MD^1,6^.

^1^*Department of Cardiothoracic, Transplant and Vascular Surgery, Hannover Medical School, Hannover, Germany.*

*^2^Department of Paediatrics, Hannover Medical School, Hannover, Germany.*

*^3^Department of Respiratory Medicine, Hannover Medical School, Hannover, Germany.*

*^4^German Center for Lung Research (DZL/BREATH), Hannover, Germany.*

*^5^These authors shared first authorship.*

*^6^These authors shared senior authorship.*

**Running title:** Impact of donor quality in lung transplantation.

**Corresponding author:**

*Fabio Ius, MD*

*Department of Cardiothoracic, Transplant and Vascular Surgery, Hannover Medical School,*

*Carl-Neuberg Strasse 1, 30625 Hannover, Germany*

*Fax +49-511-532-8446, Tel +49-511-532-2125, E-mail: [ius.fabio@mh-hannover.de](mailto:ius.fabio@mh-hannover.de).*

**METHODS**

**Calculation of PGD grade**

PGD grade in our study was calculated as follows. The paO_2_/FiO_2_ ratio was calculated using arterial blood gases taken from the right radial artery at standardized intervals. The time at the end of surgery was considered the reference time point. Then, paO_2_/FiO_2_ ratios were calculated at 24-, 48-, and 72 hours after the reference time point, and the timely corresponding chest X-rays were evaluated. At our institution, several radiologists are involved in the interpretation of post-transplant chest X-rays. However, they are highly experienced in evaluating chest X-rays after lung transplantation and adhere to standardized protocols, ensuring a consistent and expert level of analysis across all cases.

An arterial paO_2_/FiO_2_ >300mmHg signified PGD grade 0 (with clear chest radiograph) or grade 1 (with infiltrates). A paO_2_/FiO_2_ of 200-300mmHg with accompanying infiltrates were graded as PGD 2 and <200mmHg classified as PGD 3. Patients requiring ECMO support after transplantation were initially all graded PGD 3. Subsequently, however, patients with primary pulmonary electively managed with veno-arterial ECMO in the early post-operative phase as a part of a developed institutional protocol, were graded according to arterial blood gases usually taken from the right radial artery, in cases where the arterial ECMO cannula was located in a femoral artery.

**SUPPLEMENTAL TABLES**

Table 1S. Multivariable binary logistic regression analysis for primary graft dysfunction, grade 3, at 72 hours after transplantation.

| **Variable** | **Univariable** | | **Multivariable** | | | |
| --- | --- | --- | --- | --- | --- | --- |
| **PGD Grade 3 at 72 hours (n=75)** | **p-value** | **OR** | | **95%CI** | **p-value** |  |
| **Categorical variables** |  |  | |  |  |  |
| Female sex | 0.010 |  | |  |  |  |
| Previous thoracic operations | 0.020 |  | |  |  |  |
| Pediatric patients (<18 years old) | 0.042 |  | |  |  |  |
| Transplant indication, COPD | <0.001 | 0.197 | | 0.051-0.762 | 0.019 |  |
| Transplant indication, pulmonary arterial hypertension | <0.001 |  | |  |  |  |
| Transplant indication, sarcoidosis | 0.082 |  | |  |  |  |
| Pre-transplant mechanical ventilation | 0.014 |  | |  |  |  |
| Pre-transplant, ICU | <0.001 |  | |  |  |  |
| Pre-transplant, ECMO as a bridge to transplantation | <0.001 |  | |  |  |  |
| Thoracotomy, sternum sparing | <0.001 |  | |  |  |  |
| Thoracotomy, clamshell | <0.001 |  | |  |  |  |
| Double lung transplantation | 0.038 |  | |  |  |  |
| Single lung transplantation | 0.020 |  | |  |  |  |
| Intraoperative support, CPB | 0.020 |  | |  |  |  |
| Intraoperative support, ECMO | <0.001 | 2.886 | | 1.334-6.246 | 0.007 |  |
| Postoperatively extended ECMO | <0.001 | 5.130 | | 2.738-9.611 | <0.001 |  |
| Lung volume reduction surgery, atypical resection | <0.001 | 4.287 | | 1.520-12.09 | 0.006 |  |
| Lung volume reduction surgery, lobar resection | 0.005 |  | |  |  |  |
| Donor age >60 years | 0.033 |  | |  |  |  |
| Donor, history of aspiration | 0.056 |  | |  |  |  |
| **Continuous variables** |  |  | |  |  |  |
| Age (years) | 0.094 |  | |  |  |  |
| BSA (m^2^) | 0.019 |  | |  |  |  |
| Ischemic time, first lung (min.) | 0.054 |  | |  |  |  |
| Ischemic time, second lung (min.) | 0.008 |  | |  |  |  |
| PRBCs, intraoperative (units) | <0.001 | 1.089 | | 1.046-1.134 | <0.001 |  |
| FFPs, intraoperative (units) | <0.001 |  | |  |  |  |
| Donor age (years) | 0.040 | 1.030 | | 1.013-1.048 | <0.01 |  |
| Donor BSA (m^2^) | 0.020 |  | |  |  |  |
| **H-L Statistic** |  |  | |  | 0.776 |  |

BMI: body mass index; BSA: body surface area; CPB: cardiopulmonary bypass; ECMO: extracorporeal membrane oxygenation; ET: Eurotransplant; FFP: fresh frozen plasma; H-L: Hoshmer-Lemeshow; ICU: intensive care unit; OR: odds ratio; PGD: primary graft dysfunction; PRBCs: packed red blood cells; Tx: transplantation.

**Table 2S. Outcomes in ECD patients stratified according to the presence of donor computed tomography (CT).**

| **Variable** | **No donor CT (n=347)** | **Donor CT, yes (n=159)** | ***p*-value** |
| --- | --- | --- | --- |
| **PGD grade 3** |  |  |  |
| At 24 hours | 22 (6.4) | 8 (5.0) | 0.558 |
| At 48 hours | 24 (7.0) | 7 (4.4) | 0.264 |
| At 72 hours | 21 (6.1) | 7 (4.4) | 0.447 |
|  |  |  |  |
| **Graft survival** |  |  |  |
| 1 year | 91 (88, 94) | 91 (87, 95) |  |
| 5 years | 71 (66, 76) | 67 (58, 76) |  |
| 7 years | 61 (55, 67) | 56 (46, 66) |  |
| 10 years | 48 (41, 55) | 49 (37, 61) |  |
|  |  |  | 0.825 |
| **Patient survival, overall** |  |  |  |
| 1 year | 92 (89, 95) | 92 (88, 96) |  |
| 5 years | 73 (68, 78) | 69 (60, 78) |  |
| 10 years | 52 (44, 60) | 52 (38, 66) |  |
|  |  |  | 0.780 |
| **Patient survival conditioned to hospital discharge** |  |  |  |
| 1 year | 96 (94, 98) | 97 (94, 100) |  |
| 5 years | 77 (72, 82) | 72 (63, 81) |  |
| 10 years | 54 (46, 62) | 54 (40, 68) |  |
|  |  |  | 0.778 |
| **Causes of death after hospital discharge** |  |  |  |
| CLAD | 47 (14.1) | 14 (9.2) | 0.131 |
| Infection | 25 (7.5) | 12 (7.9) | 0.882 |
| Malignancy | 12 (3.6) | 3 (2.0) | 0.255 |
| Cardiac | 9 (2.7) | 4 (2.6) | 0.614 |
| Other | 15 (4.5) | 6 (3.9) | 0.780 |
|  |  |  |  |
| **Freedom from CLAD*** | (n=329) | (n=148) |  |
| 3 years | 78 (74, 82) | 84 (76, 92) |  |
| 5 years | 64 (58, 70) | 74 (65, 83) |  |
| 7 years | 57 (51, 63) | 70 (60, 80) |  |
| 10 years | 50 (42, 58) | 67 (55, 79) |  |
|  |  |  | 0.044 |

CLAD: chronic lung allograft dysfunction; CT: computed tomography.

*477 (94%) patients were considered for CLAD analysis.

Values are expressed as mean % (95% confidence interval, CI) for survival results, median (IQR), or N (%).

**Table 3S. Outcomes in ECD patients stratified according to donor age.**

| **Variable** | **Donor age ≤55 (n=258)** | **Donor age**  **>55 (n=248)** | ***p*-value** |
| --- | --- | --- | --- |
| **PGD grade 3** |  |  |  |
| At 24 hours | 19 (7.4) | 11 (4.4) | 0.160 |
| At 48 hours | 18 (7.1) | 13 (5.2) | 0.397 |
| At 72 hours | 19 (7.4) | 9 (3.6) | 0.065 |
|  |  |  |  |
| **Graft survival** |  |  |  |
| 1 year | 89 (85, 93) | 93 (90, 96) |  |
| 5 years | 66 (60, 72) | 75 (69, 81) |  |
| 7 years | 58 (51, 65) | 62 (54, 70) |  |
| 10 years | 43 (33, 53) | 53 (45, 61) |  |
|  |  |  | 0.069 |
| **Patient survival, overall** |  |  |  |
| 1 year | 90 (86, 94) | 94 (91, 97) |  |
| 5 years | 68 (62, 74) | 76 (70, 82) |  |
| 10 years | 46 (36, 56) | 57 (49, 65) |  |
|  |  |  | 0.060 |
| **Patient survival conditioned to hospital discharge** |  |  |  |
| 1 year | 95 (92, 98) | 97 (95, 99) |  |
| 5 years | 73 (67, 79) | 78 (72, 84) |  |
| 10 years | 48 (38, 58) | 58 (50, 66) |  |
|  |  |  | 0.234 |
| **Causes of death after hospital discharge** |  |  |  |
| CLAD | 26 (10.7) | 35 (14.5) | 0.211 |
| Infection | 25 (10.3) | 12 (5.0) | 0.027 |
| Malignancy | 9 (3.7) | 6 (2.5) | 0.436 |
| Cardiac | 6 (2.5) | 7 (2.9) | 0.773 |
| Other | 12 (4.9) | 9 (3.7) | 0.509 |
|  |  |  |  |
| **Freedom from CLAD*** | (n=238) | (n=239) |  |
| 3 years | 82 (76, 88) | 78 (72, 84) |  |
| 5 years | 70 (63, 77) | 64 (57, 71) |  |
| 7 years | 62 (54, 70) | 59 (51, 67) |  |
| 10 years | 57 (48, 66) | 52 (43, 61) |  |
|  |  |  | 0.401 |

CLAD: chronic lung allograft dysfunction.

*477 (94%) patients were considered for CLAD analysis.

Values are expressed as mean % (95% confidence interval, CI) for survival results, median (IQR), or N (%).

**SUPPLEMENTAL FIGURE LEGENDS**

**Figure 1S.** Figure 1S shows the Eurotransplant score and the weights of each donor variable used to calculate it.

**Figure 2S.** Figure 2S reports the graft survival (A) and the freedom from CLAD (B) with 95% confidence intervals, in patients transplanted with donor lungs showing ET scores of 6 (red line), of 7 and 8 (blue line) and between 9 and 13 (yellow line). In figure 1B, 1,409 (93.1%) patients (ET score 6, group 1, n=262; ET score 7-8, group 2, n=669; ET score 9-13, group 3, n=478) had at least 2 spirometric recordings for calculation of baseline and a survival of more than 90 days, and thus were considered for CLAD analysis.
