# Supplementary figures and images for: Impact of donor organ quality on recipient outcomes in lung transplantation: 14-Year single-center experience using the Eurotransplant lung donor score
Source: JHLT Open. 2024 Oct 11;6:100166. doi: 10.1016/j.jhlto.2024.100166 (PMC11935426; doi:10.1016/j.jhlto.2024.100166)

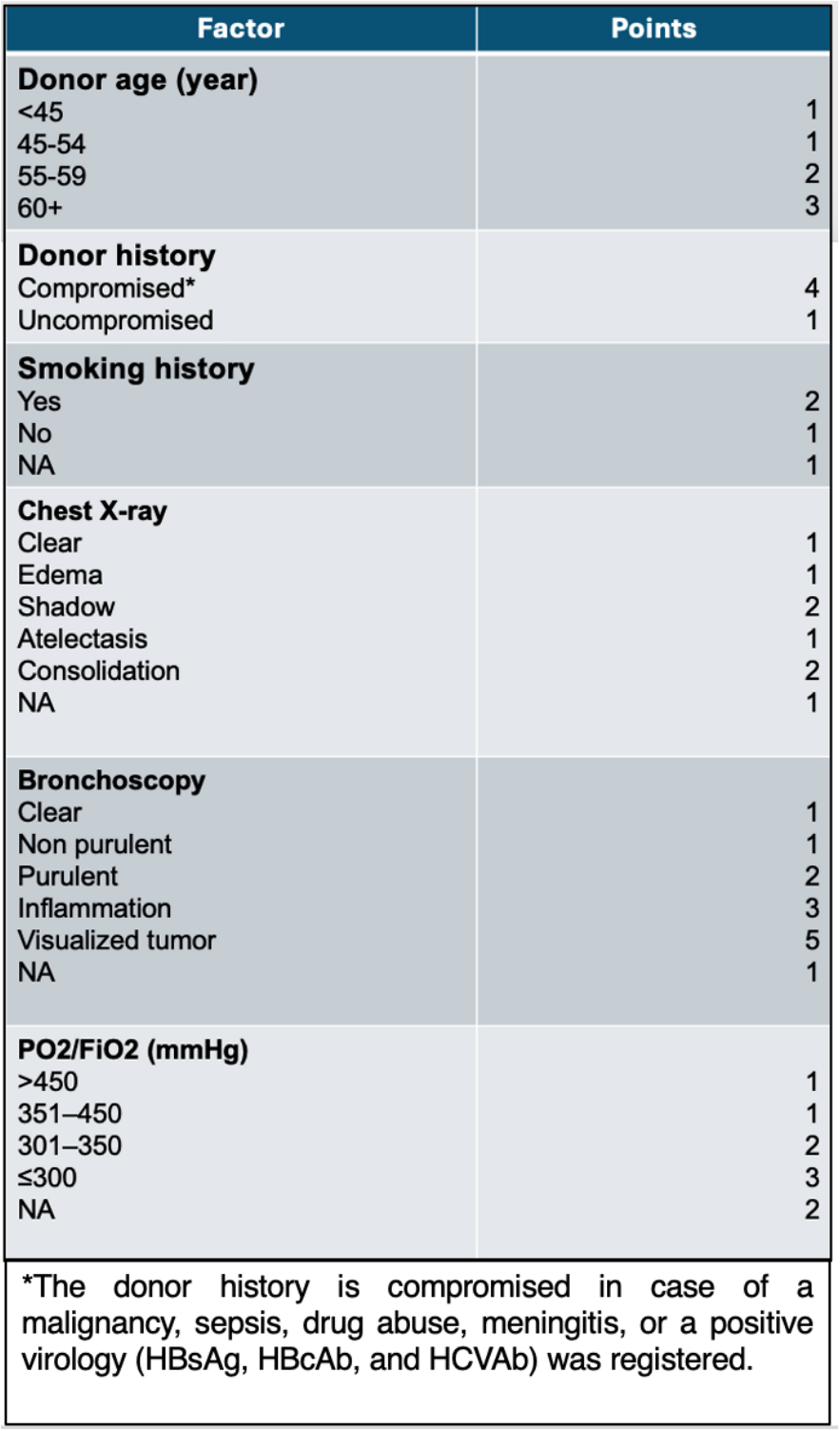

Supplement: Supplementary file 2 — Supplementary material. [file mmc2.jpg]

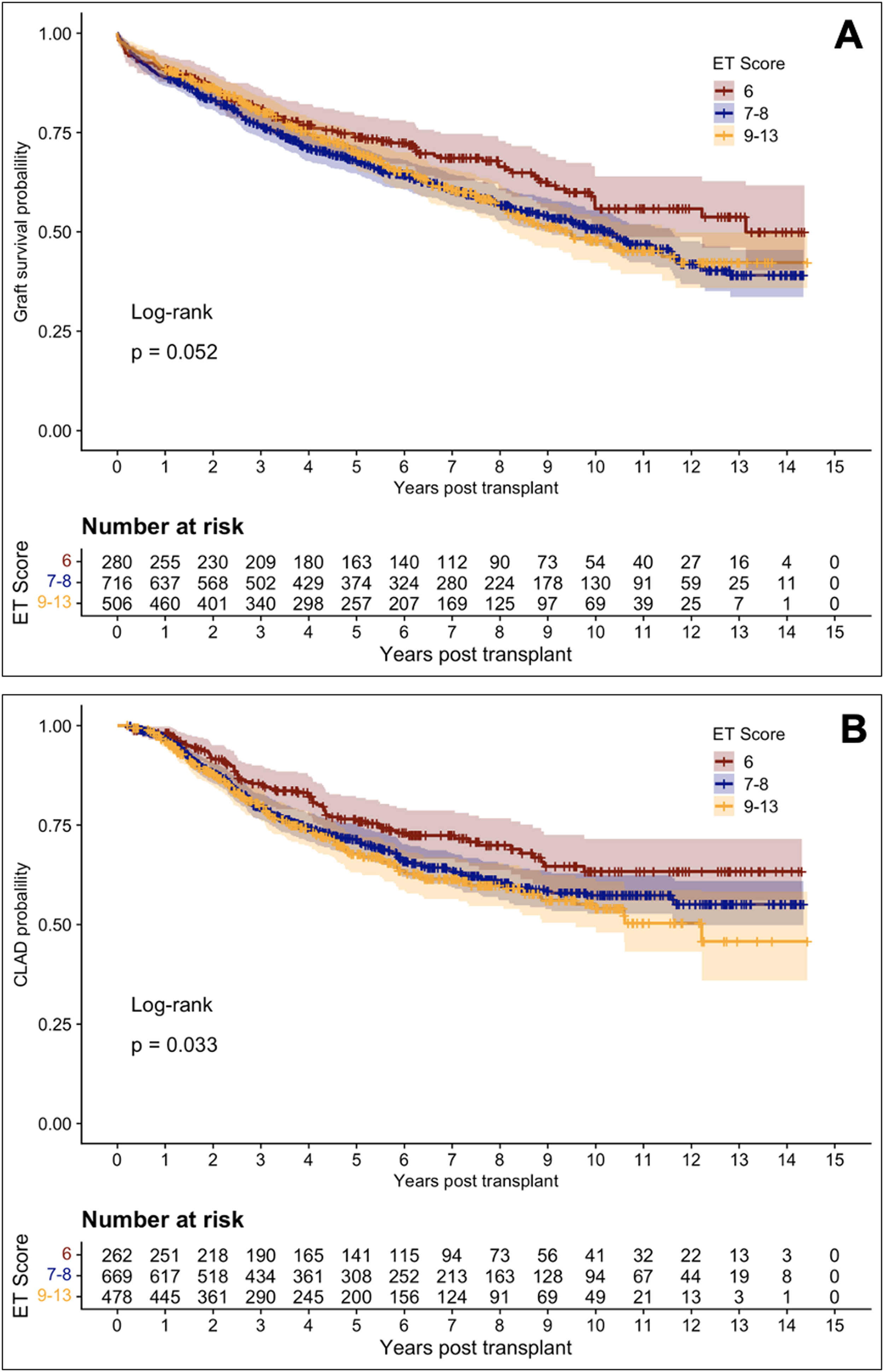

Supplement: Supplementary file 3 — Supplementary material. [file mmc3.jpg]
